# Supplementary material for: Mitochondrial Pseudogenes Suggest Repeated Inter-Species Hybridization among Direct Human Ancestors
Source: Genes (Basel). 2022 May 1;13(5):810. doi: 10.3390/genes13050810 (PMC9140377; doi:10.3390/genes13050810)
Supplement: Supplementary file 1 [file genes-13-00810-s001.zip › genes-1646051-supplementary.pdf]

Mitochondrial Pseudogenes Suggest Repeated Inter-species Hybridization in Hominid Evolution

SUPPLEMENTAL NOTES

Konstantin Popadin, Konstantin Gunbin, Leonid Peshkin, Sofia Annins, Zoe Fleischmann, Melissa Franco, Genya Kraytsberg, Natalya Markuzon, Rebecca R. Ackermann, Konstantin Khrapko

Suppl. Note SN1.

We have discovered a portion of the Ps5 pseudogene in an early pseudogene screen (Li-Sucholeiki et al., 1999). Later we located this pseudogene in the human genome and extended it to its full length using mtDNA homology criterion. As high quality gorilla and chimpanzee genomes became available (earlier versions did not contain ps5 pseudogenes or contained only their parts) we eventually recovered the entire ~9kb ps5 pseudogene sequences for each of the three species, at homologous chromosomal positions. Orangutan does not carry a homolog of this pseudogene and the corresponding chromosomal position is unremarkable.

Ps11 and ps7 pseudogenes were selected manually using BLAT search. Our vague criteria were: a) significant divergence from the human mtDNA (comparable to chimpanzee or greater), b) the availability of the pseudogene sequence in at least one great ape genome in addition to the human genome, and c) much higher similarity between pseudogene sequences of different ape species compared to their similarity to the mtDNA of the same species. This latter criterion guaranteed the existence of a long the "pseudostem" and thus implied the post-speciation gene transfer. We hope that a more systematic search for pseudogenes with unusual topologies of their joint phylogenic trees with mtDNA will reveal more instances of hybridization-like events in human evolution.

Suppl. Note SN2.

How Fig. 1, 3 and 4 were made. Tree building methods: ML, MrBayes.

Multiple alignments of nucleotide sequences were made using MAFFT (version …). The phylogenetic trees were reconstructed using two alternative approaches: the maximum likelihood approach (PhyML v. 3.0 20120412), and the Bayesian approach (MrBayes v3.2.1). GTR nucleotide substitution mode and six categories of gamma distribution of site variability values were used in both approaches.

Suppl. Note SN3.

ML/ Jackknife divergence estimation.

[How fig2 was made].

Mitochondrial genomes of 83 sequences of human, chimpanzee, gorilla and orangutan mtDNA [1] combined with two mitochondrial genomes of gibbon and baboon (NC_002082.1 and Y18001.1; outgroup), two mitochondrial genomes of extinct humans (NC_023100.1 and NC_013993.1) and two Ps5 pseudogenes from nuclear genomes of

humans, and gorillas were aligned using SATe v. 2.2.7 program. The alignment was trimmed to the region covered by the pseudogenes Ps5. The final alignment was 9171bp long. This alignment was subjected to the 5000 iterations of the standard jackknife procedure: randomly selected 4585 alignment positions were used to reconstruct the phylogenetic tree using RAxML v. 8.0.12 (options: -f d -m GTRGAMMA) program. To perform the summation of branch lengths from 5000 jackknifed trees, a Perl script was written. This script extracted the information on the topology ('consensus' or 'alternative') and branch lengths of each jackknifed tree. The variances of length of selected branches indicated on Fig 2. The 'consensus' topology (~ 93% from all cases) shown on Fig 1; this topology clustered Ps5 pseudogenes with the most recent common ancestor (MRCA) of humans, chimpanzees and gorillas. The alternative topology (~ 6% from all cases) clustered Ps5 pseudogenes with gorillas only. The tree branch lengths obtained using this alternative topology were excluded from our analysis because the GTR matrices corresponding to alternative tree topology differ significantly from the matrices obtained based on 'consensus' tree topology which is also statistically optimal (see **Suppl. Note SN2**). Moreover, the alternative topology was non-parsimonious and clearly included reverse mutations.

This procedure resulted in an estimate of 6±1% for the divergence of the ps5 stem. The results of the jackknife procedure were represented as frequency distributions (Figure 2). For the ps5 stem, the divergence was reduced by 25% to account for the 25% of mutations of the ps5 stem that are of pseudogene origin (**Suppl Note SN5**) this yielded the corrected estimate 4.5 ±0.8% divergence.

Suppl. Note SN4.

Stability of the tree topology and branch lengths.

To test the presence of alternative topologies of trees (figures 1, 3 and 4) reconstructed on the basis of full-length alignments we used CONSEL v. 1.20 2011/05/12 program package. Alternative tree topologies were generated using 50 random starting tree topologies by PhyML v. 3.0 20120412 (options: -o tlr -c 6 --n_rand_starts 50 -s SPR --print_site_lnl). Trees shown on the figures 1, 3 and 4 reflects optimal tree topologies, which are statistically significant (AU, KS, SH tests, nonparametric bootstrap) better (p<0.01) than any alternative topologies under three different evolutionary models considered (GTR, HKY, TN) and various outgroups (with and without orangutan, baboon, gibbon, macaque).

To test the presence of alternative topology of trees (figures 1, 3 and 4) reconstructed on the basis of sequestered alignments we use 25% and 50% jackknifing as described in (**Suppl. Note SN3)**. In order to obtain consensus phylogenies from jackknife iterations we used sumtrees.py program from dendropy v. 3.3.1. The same consensus topologies as shown on figures 1, 3 and 4 with >80% clade support at every inner node were obtained. Additionally, we trimmed the 9k-long alignment of Ps5 to approximately 2.5k and reconstructed trees using PhyML v. 3.0 20120412 and MrBayes v3.2.1 (GTR nucleotide substitution mode and six categories of gamma distribution of site variability values were used). The same topology (as shown on figure 1) was obtained after such an enormous alignment trimming and using various outgroups (with and without orangutan, baboon, gibbon,

macaque). Thus, the topologies of trees shown at figures 1, 3 and 4 are robust to changes in evolutionary models and to vast changes fragment length.

Test for the pseudogene branch length bias. We were particularly concerned whether the extraordinary length of the ps5 stem, which is the main finding of this study, could be a result of some kind of unforeseen artifact caused by the tree building algorithms. We have demonstrated that the use of various algorithms, parameter sets, and the jackknife procedure do not significantly affect the relative length of ps5 stem. Still there is a possibility that there was a parameter that we failed to pinpoint that was responsible for artefactual extension of the stem. A trivial example of such a situation would be the use an algorithm with a molecular clock, which would naturally extend ps5 stem to keep it leveled with other branches. We reasoned that from the algorithmic point of view, the only detectable difference between the pseudogene subtree and other branches of the phylogenic three (such as gorilla, and human/chimp) is the overall shorter length if the pseudogenic branches, which is a result of the slower mutational rate of the nuclear DNA sequences. If so, this shorter length is *the only possible source of algorithmic bias* with respect to the assigning of the branch lengths. If such a bias was present in the building of the trees used in this study, then *artificial increase* of the pseudogene branch lengths should result in the decrease of such a bias. To test this hypothesis, we introduced random mutations (3%, transitions only, to mimic the mtDNA mutational bias) into the sequences of the ps5 pseudogenes (human and gorilla) and reconstructed the trees. The result is shown in the figure below. As expected, mutations resulted in longer outer pseudogene branches, the ps5 stem, however did not shorten, which strongly falsifies the algorithmic bias hypothesis.

Suppl. Note SN5. The proportion of "mitochondrial" vs "pseudogenic" mutations in the ps5 stem (and the stems of ps11 and ps7).

To explore the mode of evolution of the ps5 locus along the ps5 stem, we examined the type of mutations comprising the ps5 stem. We determined the proportion of synonymous and non-synonymous mutations that happened along that stem and compared this ratio to those in normal mtDNA branches ("mitochondrial" mutations) and those in outer branches of the ps5 subtree ("pseudogenic" mutations). To estimate the proportion of "mitochondrial" mutations in the ps5 stem (**Figure 1**), we used a linear approach. We assumed that mutations of the ps5 stem were a mixture of mitochondrial and pseudogenic ones. We then determined the proportion of the mitochondrial and the pseudogenic mutations in the stem that would yield the correct value of average synonymicity/GERP score for the mutations of the ps5 stem.

First, we created lists of mutations that have occurred unambiguously on specific sub-trees of the great ape tree. To do so, we aligned the appropriate fragments of the following mitochondrial genomes: two diversified humans (contemporary human NC_012920.1 and ancient human from Denisova cave NC_013993.1), two gorilla species (Gorilla beringei graueri A929_Kaisi; Gorilla gorilla gorilla 9749_Kowali), two chimpanzee species (Pan paniscus 9731_LB502; Pan troglodytes troglodytes A957_Vaillant), two orangutan species (Pongo abelii A947_Elsi; Pongo pygmaeus A944_Napoleon), the two 9kb long

ps5 pseudogene sequences (human and gorilla), and the gibbon (NC_002082.1 Hylobates lar) as an outgroup. In the alignment, we identified base pair changes that occurred uniquely in a single sequence or several sequences belonging to the same clade. For example, the unique mutations of the Homo clade are changes that were observed either only in the human, only in the Denosovan sequence or in both *and only there*. The numbers of unique mutations in each species/clade are shown in table S1. We then determined the type (synonymous/non-synonymous) of each unique mutation. While in most cases the type of unique substitutions was unambiguous, in rare cases it was not, because the ancestral nucleotide was unknown. To resolve such cases, we selected 5 species (gibbon; Pongo pygmaeus, Gorilla beringei, Pan paniscus and ancient human) as a representative set of references used to determine the type of unique substitutions: non-synonymous or non-coding/synonymous. If the mutation was non-synonymous in at least one outgroup, we considered this mutation as non- synonymous, otherwise the mutation considered as synonymous or non-coding. The resulting fractions of non-synonymous mutations in each species/clade that we considered are listed in Table S1.

To estimate the fraction of “mitochondrial” mutations in ps5 stem quantitatively, we noted that mutations of the ps5 stem must have been accumulating in functional mtDNA for some time, and, then after mtDNA was inserted into nuclear DNA, continued to accumulate in the pseudogene. Thus, mutations of the ps5 stem constitute a mixture of the “mitochondrial” and the “pseudogenic” mutations. As expected, the “mitochondrial” and the “pseudogenic” mutations differ considerably with respect to the fraction of non-synonymous mutations. Indeed, the fraction of NS mutations in the mitochondrial and pseudogene branches differs greatly (average 0.26 vs. 0.87*- see **Table S1** rows A and B, and the “comment” at the end of Note) and statistically significantly ($p<0.00001$, Mann-Witney test on the 50% jackknife dataset). We then determined, using simple linear equation, the proportion of the “mitochondrial” mutations which yielded the observed overall fraction of non-synonymous mutations in the ps5 stem.

The above estimate critically depends on the proportion of non-synonymous mutations in mtDNA used as reference. We appreciate that this proportion differs significantly between different species/branches (Table 1S, row B) and this variation is a major source of variance in our estimates. To account for this variance, we calculated the apparent fraction of “mitochondrial” mutations in the ps5 stem using different mitochondrial branches as reference, i.e. Human, Chimpanzee and Gorilla, each from the split with the other apes to the branch tips. In doing so we tried to find a reference that is most similar to the ps5 stem in length/number of mutations and belongs to the same group (hominines). We included mutations from the terminal branches because the mtDNA precursor of the ps5 was actually a terminal the branch when it became “frozen” in the nuclear DNA environment. To estimate the deviation of our estimate, we considered estimates based on different mitochondrial branches as independent measurements. We did so because the variance among different branches appears to be the main source of variance in this estimate. As seen in Table S1 cells B3 and B4, the estimated fraction of “mitochondrial” mutations is about 0.75 with a reasonably tight formal standard deviation. In other words, there are 25% pseudogenic mutations in the ps5 stem.

Tests for bias: Because the estimate of the proportion of the "Mitochondrial" mutations in the ps5 stem depends strongly on the fraction of NS mutations in the mtDNA used as a reference, we strived to make sure that our selection of reference mitochondrial branches did not impose any bias on our estimate, and in particular that our estimate remained conservative.

First, we tested whether limiting reference branches to the hominine mtDNA only could have resulted in an overestimation of mitochondrial mutations in ps5 stem. We therefore additionally included branches leading to the twoorangutan species from their common ancestor (Table S1.C). As shown in Table S1, inclusion of orangutan branches *increases* the estimate, so our estimate is indeed conservative.

Second, we reasoned that the apparent proportion of NS mutations in a branch segment may depend on how deep in the tree that segment is located. In general, one expects higher proportion of NS mutations on deeper branches, because NS mutations have a lower chance to be obscured/saturated by reverse mutations. Hominine phylogenic tree offers very limited number of possible reference mtDNA.

branches, all of which we have used. Our reference branches are slightly deeper than the ps5 stem, so we were concerned whether this difference might have imposed a positive bias on our estimate. We therefore used as a control a set of shallower reference branches, i.e., the terminal hominine *subspecies* branches (Table S1.D). Reassuringly, the resulting estimate is essentially the same as with the deeper reference branches. We conclude that the effect of saturation of synonymous mutations at the branch level where we are working is not significant enough to bias our estimate.

We further explored the same question using Bayesian reconstruction of ancestral sequences. The various stringency settings used in reconstruction resulted is various estimates, all of which are lower than 25% pseudomutations in the ps5 stem. In conclusion, our estimate of 25% pseudogenic mutations in ps5 stem using unique mutations is indeed conservative, and we are comfortable to use it in further analysis.

What are the implications of the presence of ~25% of pseudogene mutations in the ps5 stem? One possibility (discussed above) is that these mutations accumulated after pseudogene insertion but before the pseudogene diverged into the three lineage(s). These mutations could have accumulated while the pseudogene was tossed around (and probably eventually fixed) in the population of the extinct hominine. If so, these mutations could have been counted towards the divergence prior to hybridization event. We have chosen not to do this because there is a possibility of an "initial burst" of mutations shortly after the insertion of the pseudogene, so the number of these mutations might be misleading as far as the evolution timing is concerned. The other possibility is that the Ps5 precursor mtDNA actually underwent efficient selection, which resulted in a higher percentage of non-synonymous mutations, which we interpreted as the presence of pseudogenic mutations. Some true mtDNA lineages indeed possess higher proportions of nonsynonymous mutations, e.g. gorilla or orangutan lineages. For example, the *Pongo abelii* terminal branch contains 37% nonsynonymous mutations, i.e. very close to the 42% value of the ps5 stem (which at the time of pseudogenization was a

terminal branch of a similar size). In either case, the correct interpretation of these mutations in terms of the evolution time is questionable, so we have chosen to keep our estimates conservative and not to count these mutations at all.

In conclusion, we favor the interpretation, that the mtDNA precursor of the pos5 pseudogene has been converted into a pseudogene at the time equivalent of the ¾ of the ps5 stem evolution time.

Comment on the NS fraction in pseudogene branches.

Note that fraction of NS mutations in the pseudogenic branches (0.87) is somewhat higher than expected for a typical neutral sequence. Higher than expected percentage of NS mutations on the pseudogene branches is most likely caused by the use of "unique mutations" i.e. those not present anywhere except the branches in question. This automatically filtered out recurrent mutations, which are expected to be more permissive and thus enriched by synonymous mutations. Another (not mutually exclusive) contribution may be related to the fact that actual somatic mutations in mtDNA are more non-synonymous than random nucleotide changes. This peculiarity is related to non-synonymity of mutational hotspots: any mutational hotspot will mutate to a less mutable nucleotide unless the change is prohibited because of non-synonymity of the change.

Table S1. Estimation of the fraction of mitochondrial mutations ps5 stem.

		Fraction of non-syn mutations in branch	Number of mutations in branch	Estimated % of mitochondrial mutations in ps5 stem	SD
A	Ps5 stem	0.42	122		
	Pseudo branches	0.87	123		
B	Homo	0.23	130	0.7	
	Pan	0.24	109	0.71	
	Gor	0.32	164	0.82	
	Average Hominine Long Branches	0.26		0.75	0.06
C	P. pyg	0.35	127	0.87	
	P. abel	0.37	137	0.90	
	All Hominine Long Branches with Pongo	0.3		0.80	0.09
D	H. sapiens	0.21	35	0.68	
	P. bonobo	0.24	43	0.71	
	P. troglodytes	0.28	59	0.76	
	G. gorilla	0.32	49	0.82	
	G. beringei	0.36	53	0.88	
	Hominine Short Outer Branches	0.28		0.77	0.08

Suppl. Note SN6. mtDNA divergence in the ancestral population. Role of population size.

To explore the possibility that a larger size of the ancestral population could have accounted for the surprising divergence of the Ps5 precursor mtDNA/Extinct Hominine, we estimated the actual mtDNA divergences in modern hominine populations and compared

these to the nuclear DNA **N*e* of the same populations** (**Figure S4 blue diamonds/line**). As a proxy of "populations", we used the formal subspecies, conservatively assuming that individuals within a subspecies are sufficiently interconnected to be considered a population. We then estimated the divergences for mtDNA sequences available for each of the subspecies in the database [1].

The divergences were estimated similarly to estimating the divergence of hominine species and genera (**Fig 2 and Supl. Note SN3**) except we did not perform the jackknife procedure. In short, we constructed an ML tree for each subspecies, found the longest branch from the most recent common ancestor of the subspecies, recorded its divergence, and plotted these divergences against the **N*e*** of the subspecies as determined in [1]. The resulting plot is remarkable in several aspects.

First, this demonstrates that the divergence of the Ps5 precursor mtDNA (red dotted line in **Fig S4**) far exceeds mtDNA divergences in genetically connected populations.

Second, to our surprise, we discovered that modern hominine populations show an inconsistent, if any, increase in mtDNA divergence with increased nuclear **N*e***. In an ideal population, the two **N*e***'s, Ne_{nuc} and Ne_{mit}, should be proportional to each other. We do not know why this is not so in hominine populations we studied; perhaps rare events of positive selection in mtDNA might stochastically decrease Ne_{mit} can ameliorate the impact of the species-specific effective populations size on mtDNA divergence ([2] or the divergence of mtDNA depends on gender-specific factors [3].

"Based on comparing patterns of genetic variation on the X chromosome and the autosomes, several recent studies have found evidence of sex-biased demographic processes during human history, often suggesting that the effective population size of females was higher than that of males throughout recent human history (Nf>Nm, if Nf represents the effective number of breeding females and Nm represents the effective number of breeding males)" (see **Suppl. Note SN7**). Whatever the mechanism, this observation does allow us to conclude that, if the ancestral population was "similar" to modern hominine populations, then tentative extrapolation to 50,000 individuals, i.e. to the **N*e*** of the HCG ancestral population (blue broken line) barely predicts any increase of mtDNA diversity compared to the contemporary populations (**Fig S4**, broken blue line, purple diamond), which supports the hybridization hypothesis.

The grand tree in Fig S1 (Mr Bayes, GTR) visually reconfirms the puzzling observation of Fig S4: the subspecies subtree depths (which essentially graphically represent divergence and/or **Ne**) are unexpectedly similar in all subspecies irrespectively of the Nuclear DNA – derive Ne from 5,000 for *T.t.verus* to 30,000 for *T.t.troglodtes*.

Suppl. Note SN7. Timing of the ps5 pseudogene insertion into the nuclear genome and of the hybridization event.

To determine the timing of the ps5 insertion we compared the position of this event on the phylogenetic tree to the position of the closest known event, i.e. the separation of the Human and Chimpanzee mtDNA lineages, which we considered to have occurred 6 My ago, following the commonly accepted number. To do so, we subtracted and

added the lengths of branches connecting the two events (green route in the Figure S5) appropriately to calculate the time difference between them concidering 1% divergence equal to 1Ma (Figure S2). Note that, following the logic of **Suppl. Note SN5**, we considered that the insertion of ps5 occurred about 75% down the ps5 common stem (at the border between the green and the blue in Figure S5). The calculations were done using two approaches: the ML (see **Suppl. Note SN3**) and MrBayes (as employed by Geneious 8.1, Biomatters). The confidence intervals of the estimates were determined using the 50% jackknife approach in the ML and 95%HPD credible intervals in MrBayes. Using the ML approach, the variances of MRCA$_{PsHCG}$-tips and MRCA$_{HCG}$–tips phylogenetic tree branch lengths were determined. These two lengths estimated as 0.106+/-0.0065 (in mean+/-2*sd notation) and 0.066+/-0.0045, correspondingly. Therefore (assuming that the coalescence time to MRCA$_{HCG}$ is approximately 6 Ma), the coalescence point of Ps5 ancestor could be estimated from 10.8 Ma to 8.5 Ma. Taking into consideration that the MRCA$_{PsHCG}$ - MRCA$_{Ps}$ branch length is 0.055+/-0.0045 and that the expected proportion of pseudogenic mutations in the ps5 stem is 25%+/-6% (Suppl. Note 6), the time of transfer of Ps5 to nucleus is 5.8 +/-2.2 Ma (ML/50% jackknife) with 95% confidence. Estimates obtained using 95%HPD credible intervals in MrBayes are similar: 6.2 +/-1.6 My ago (MrBayes, 95%HPD). Thus, we conclude that ps5 insertion happened approximately at the time of Human/Chimpanzee separation about 6 Ma.

Note that the number 6Ma is merely a relative measure. It entirely depends on our choice of the measuring scale, that is, the timing of the human/chimpanzee divergence. In fact, we merely state that hybridization happened during the epoch of human/chimpanzee split.

Of note, the time of pseudogene insertion is only the earliest time limit for the hybridization event. The ps5 pseudogene might have been evolving in the extinct hominine population for an uncertain time after the insertion, prior to hybridization. Indeed, ~25% of the mutations of the common ps5 stem that are "pseudogenic" could have accumulated during this period. However, the rate of accumulation of these mutations is not known. They might have potentially accumulated during an initial mutational burst soon after the insertion (see Suppl Note SN5 for more details). We therefore have chosen to make a conservative estimate of the time of hybridization, assuming the it happened immediately after the pseudogene insertion. However, we cannot exclude the possibility that the hybridization happened much later.

Suppl. Note SN8. mtDNA divergence in the ancestral population. Role of the "population structure".

The peculiar observation (**Suppl. Note SN6**) that in modern hominine populations, mtDNA divergence shows weak (if any) proportionality to the nDNA **N*e*** may mean that mtDNA divergence is primarily driven by some other, presumably mtDNA/gender-specific factors. One possibility is a barrier to the gene flow of mtDNA but not of nDNA.

For example, the disproportionally high mtDNA diversity in the *P. t. verus* (compared to its low nuclear DNA **N*e***, rightmost point in

Fig. S4) could be related to some physical boundaries, such as a river, between cryptic subpopulations [4]. Such a barrier could have been regularly crossed by males in search for sexual partners or unclaimed territory, but not by females who have other aspirations. Decreased mtDNA gene flow between the subpopulations that are otherwise genetically well connected (as far as nDNA is concerned) by the wandering males could have resulted in an excess divergence (relative to that expected in an ideal population) of the mtDNA. In contrast, relatively lower mtDNA diversity in *P. t. troglodytes* could be related to overall higher mobility of the females (and thus mtDNA) within the population because of female exogamy that these apes are known to practice. These inferences are corroborated by evidence from outside the Homininae subfamily. *Pongo abelii* shows an unusually high mtDNA diversity (yellow diamond in **Fig. S4**), which may be related to the fact male/female motilities in this species are similar if not reversed.

Of note, such gender mobility biases may not be strong enough to measurably affect X-chromosome gene distribution, because the flow of X chromosomes is only decreased 2-fold, but not potentially entirely prevented by such a barrier, as in case of mtDNA.

Here, it would be relevant to mention the naked mole rat as an ultimate case of female immobility leading to huge mtDNA diversity relatively to that of nDNA. Indeed, intra-species mtDNA divergence in the naked mole rat appears to greatly exceed, e.g., the ps5 precursor divergence, while nDNA is unusually uniform [5]. This implies that, in general, mtDNA diversity appears to be difficult to predict. However, mtDNA divergence in hominines appear to be rather low, even in populations with high **N*e***. Thus, as long as the hominine ancestral population had a population structure similar to that of the contemporary hominines, the divergence of the "pseudogene precursor mtDNA" can be considered rather extraordinary and supports an introgression of a rather distant species. If, however, the structure of the ancestral population was significantly different , for example, if there were strong boundaries to female mobility, male exogamy, or similar – then "pseudogene precursor mtDNA" could have evolved in an otherwise continuous population. The presence of such a population structure would be an interesting discovery, perhaps not less interesting than a discovery of an interspecies hybridization. What it suggests is that the ancestral condition for hominines (and the other ape lineages) is not a chimpanzee-like one. This would contradict the **Copeland et al** isotopic study of South African later hominins that showed female exogamy [6].

Supplementary Figures

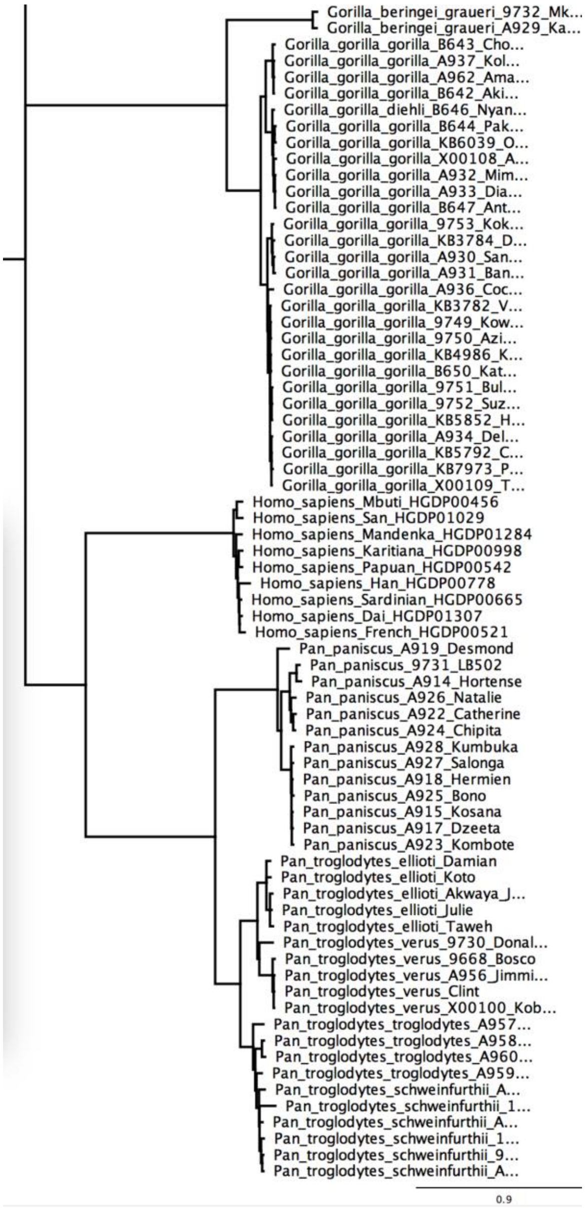

Figure S1. Grand tree of Human, Chimpanzee, and Gorilla clades, built by Mr. Bayes.

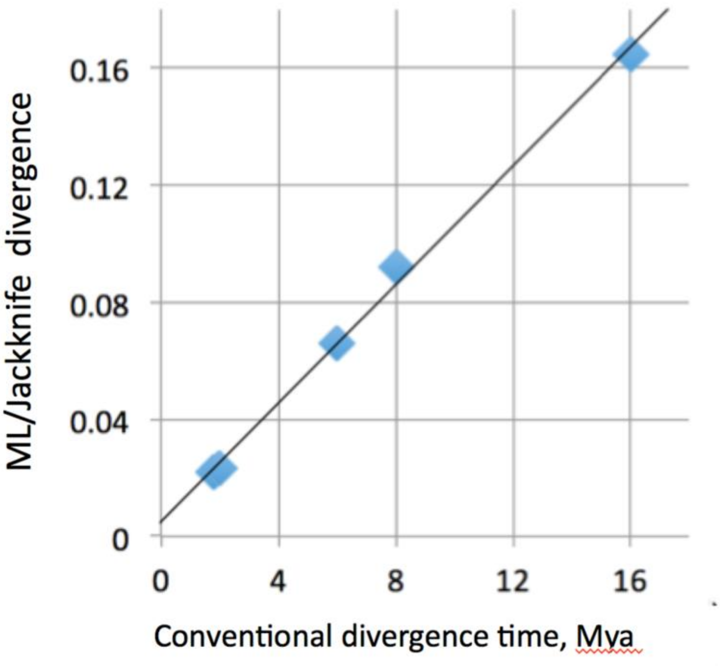

Figure S2. ML/ Jackknife divergence estimation is approximately proportional to the conventional divergence times (Orangutan – 16Mya, Gorilla – 8Mua, Chimpanzee – 6Mya, Chimp/Bonobo 2Mya, West/east gorilla – 1.7 Mya). About 1% ML/ Jackknife divergence corresponds to 1My.

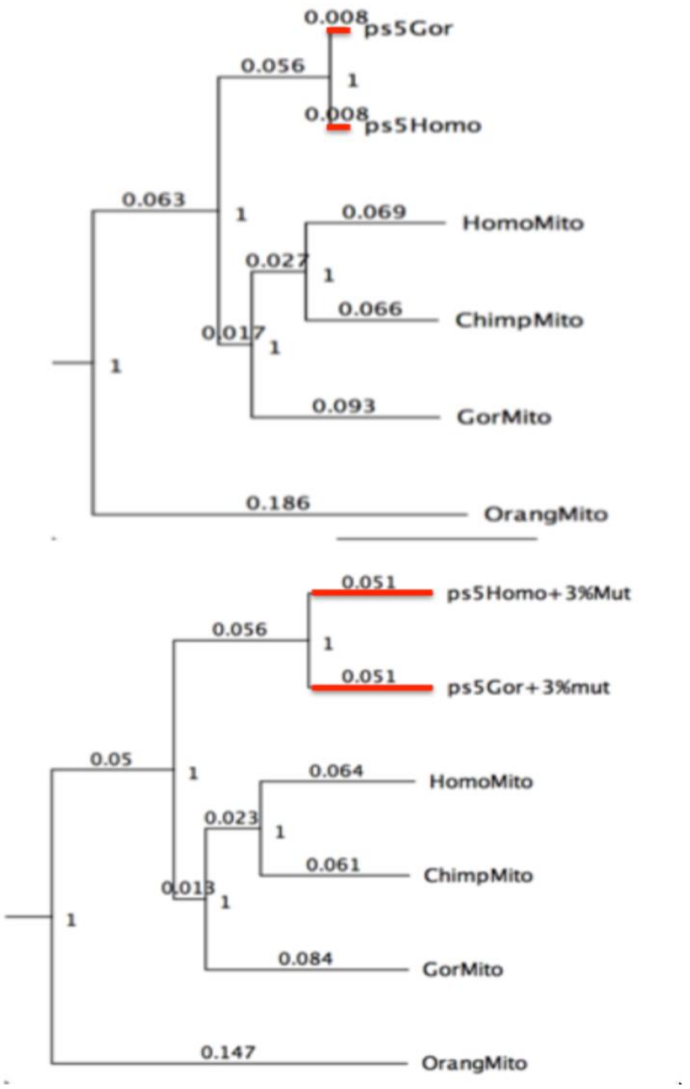

Figure S3. Test for the pseudogene branch length bias. Artificial lengthening of the pseudogene branches (added 3% transitions, lower image)) does not affect the ps5 stem length and overall topology/topography of the tree.

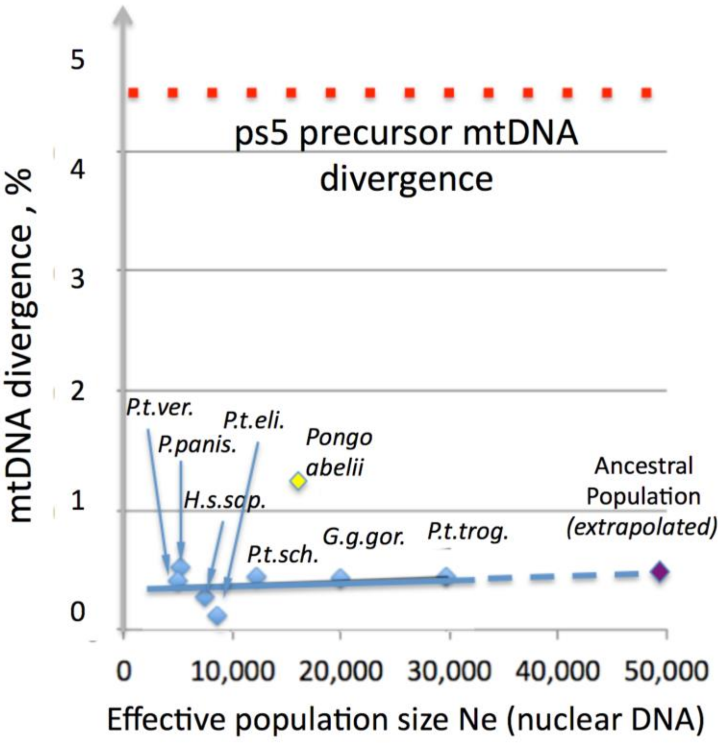

Figure S4. The divergence of mtDNA from the MRCA of each population/subspecies as a function of the effective population size Ne_{nuc} of the living hominine population/subspecies (blue diamonds). Ne_{nuc} data are from [1]. Note the divergence of the s5 precursor mtDNA (red dotted line on top of the plot) shown for comparison. See text for details.

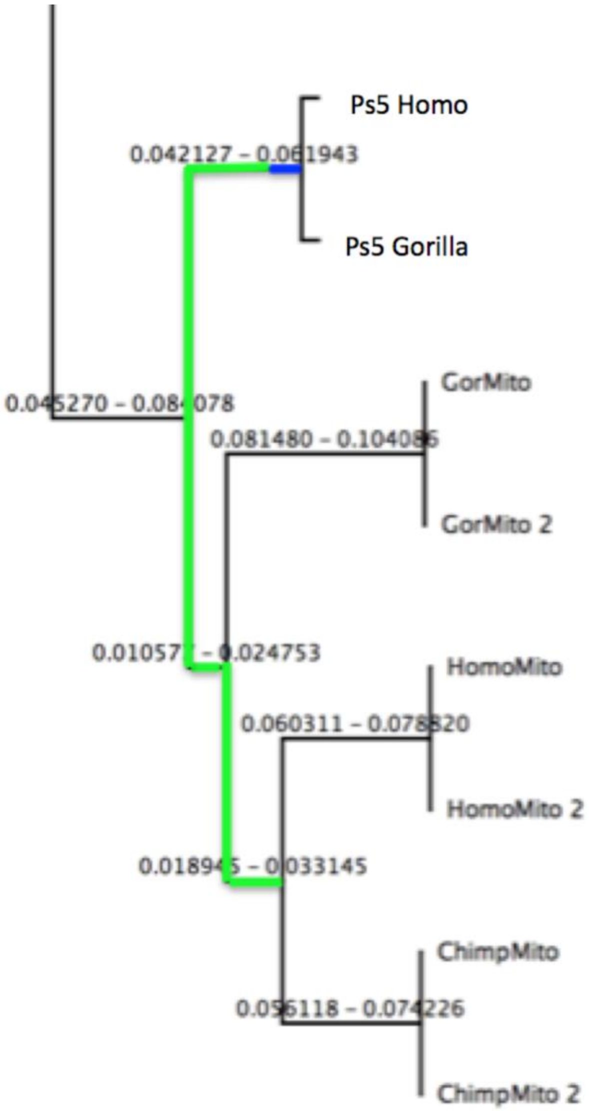

Figure S5. A Bayesian tree used to calculate the time of ps5 pseudogene insertion into the nucleus. The path used to calculate the time interval between Human/Chimp separation (6My) and ps5 transfer is sown in green. Blue is the presumed pseudogenic portion of the ps5 common stem. 95%HPD intervals used to calculate the error of the estimate are shown by the corresponding branch segments (duplicates were used to force Geneious to display 95%HPD intervals, apparently a software glitch).

Supplementary References:

1. Bazin, E., Gléminand, S., and Galtier, N. Population Size Does Not Influence Mitochondrial Genetic Diversity in Animals. *Science*, **2006**, 312, 570–572.
2. Wilson Sayres, M.A., Lohmueller, K.E., Nielsen, R. Natural Selection Reduced Diversity on Human Y Chromosomes. *PLOS Genetics*, **2014**, *10*, e1004064.
3. Eriksson, J., Hohmann, G., Boesch, C., and Vigilant, L. Rivers influence the population genetic structure of bonobos (Pan paniscus). *Mol. Ecol.*, **2004**, *13*, 3425–3435.
4. Faulkes, C.G., Abbott, D.H., O'Brien, H.P., Lau, L., Roy, M.R., Wayne, R.K., Bruford, M.W. Micro- and macrogeographical genetic structure of colonies of naked mole-rats Heterocephalus. *Mol. Ecol.*, **1997**, *6*, 615–628.
5. Copeland, S.R., Sponheimer, M., de Ruiter, D.J., Lee-Thorp, J.A., Codron, D., le Roux, P.J., Grimes, V., and Richards, M.P. Strontium isotope evidence for landscape use by early hominins. Nature, **2011**, *474*, 76–78.
